# Supplementary material for: A deep-learning-based RNA-seq germline variant caller
Source: Bioinform Adv. 2023 Jun 13;3(1):vbad062. doi: 10.1093/bioadv/vbad062 (PMC10320079; doi:10.1093/bioadv/vbad062)
Supplement: vbad062_Supplementary_Data [file vbad062_supplementary_data.zip › Cook_et_al_Supp_Figures.pdf]

Supplementary Figures

Supplementary Figure S1

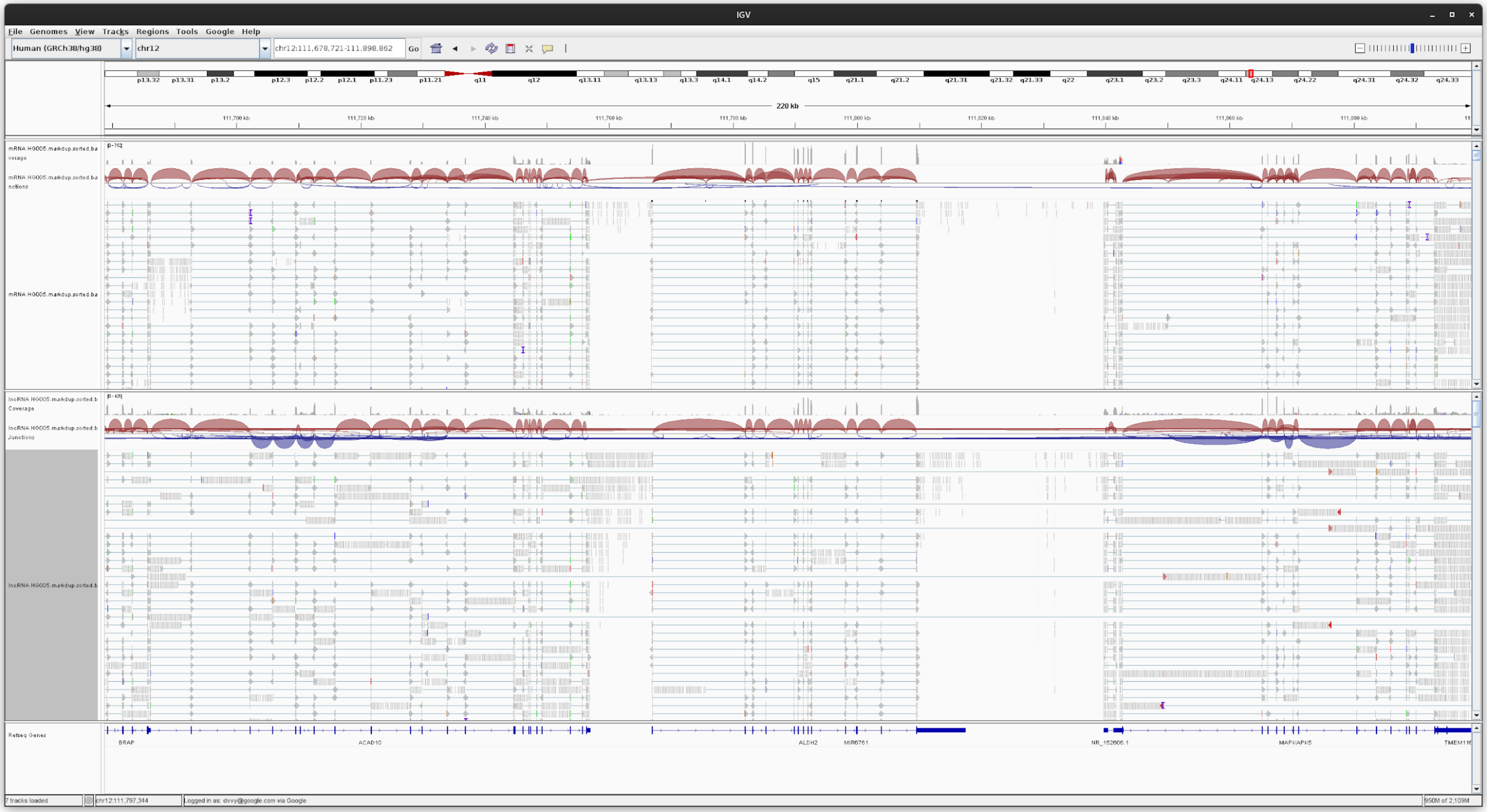

Supplementary Figure S1: Visualization of RNA-seq data alignments. A screenshot of the Integrative Genomics Viewer (IGV) showing skip regions present in RNA-seq data.

Supplementary Figure S2

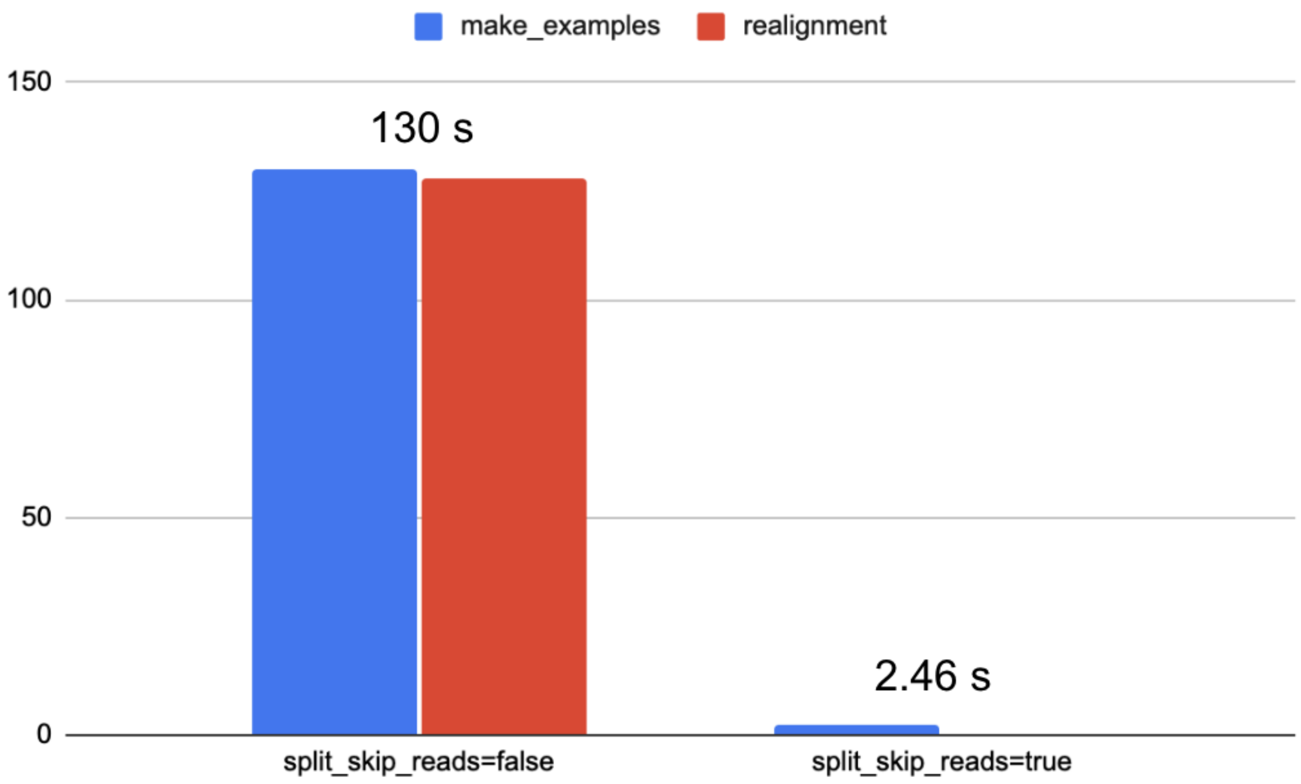

Supplementary Figure S2: The impact of split_skip_reads flag in example generation time. The time required to make examples and perform realignment is shown in seconds for chromosome 1:1-100kb. Setting `--split_skip_reads` significantly reduces the time required to perform local-realignment when processing RNA-seq data.

Supplementary Figure S3

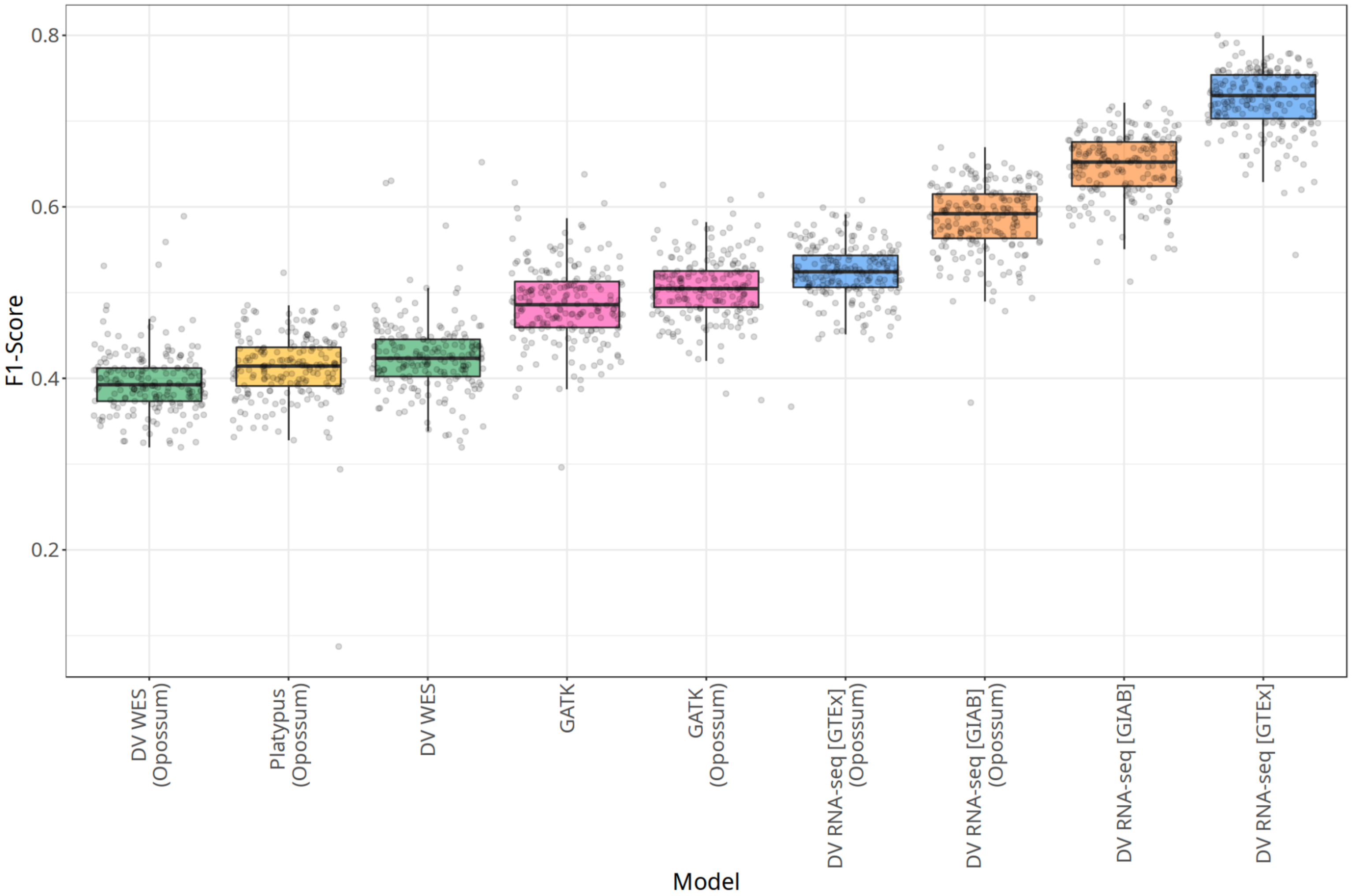

Supplementary Figure S3: INDEL F1-scores in CDS regions for 200 RNA-seq samples. INDEL F1 scores in CDS regions are shown for 200 GTEx RNA-seq samples across Platypus, GATK, DeepVariant WES, and DeepVariant RNA-seq. Data that was pre-processed with Opossum is labeled as such.

Supplementary Figure S4

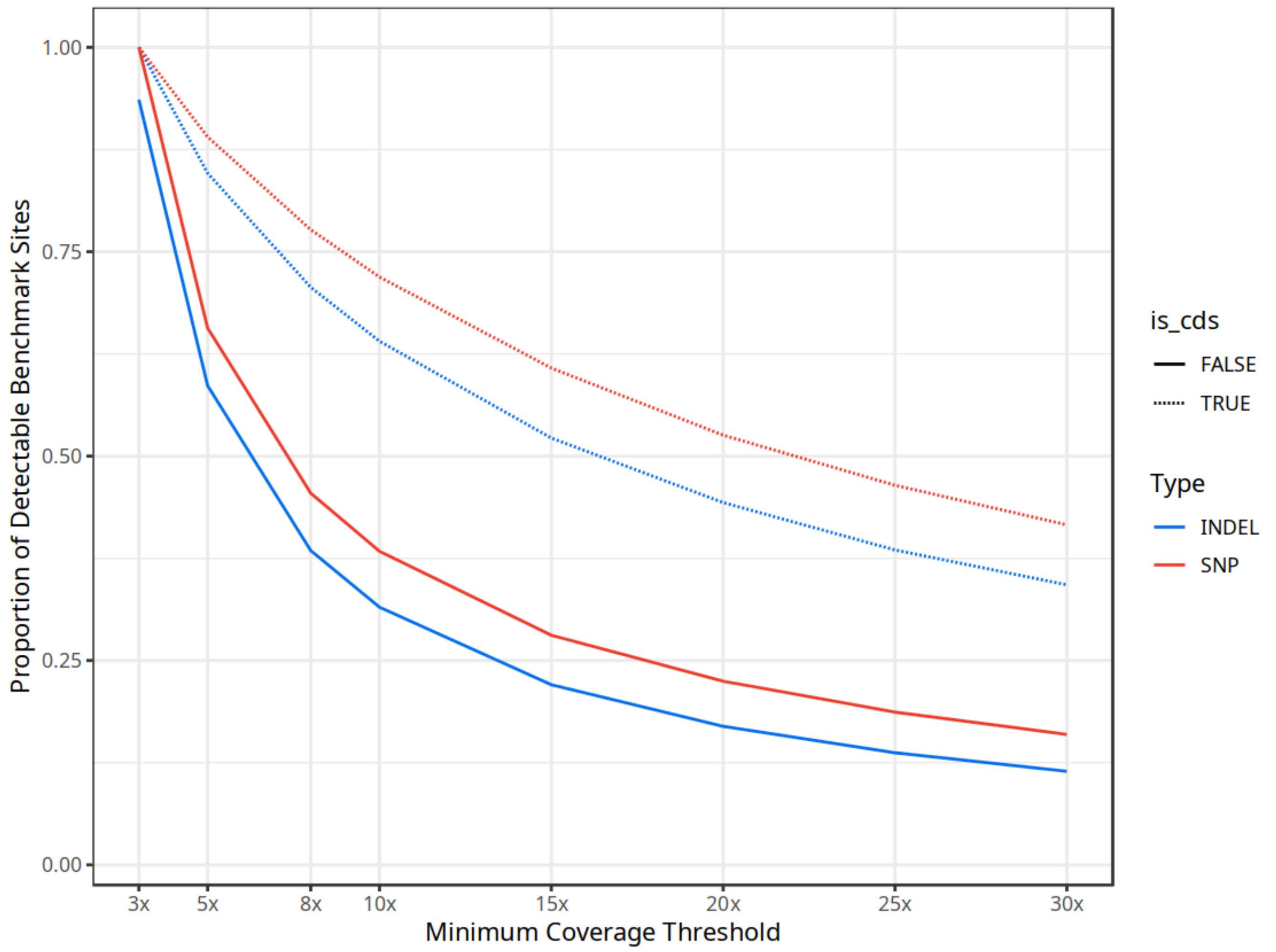

Supplementary Figure S4: Proportion of detectable sites. The proportion of detectable benchmark sites are shown across minimum coverage thresholds. "Detectable" sites are label sites present within 3x regions that remain at higher coverage thresholds. Curves are shown for SNP and INDEL variants present within or outside of CDS regions. These curves show how the proportion of 3x variant sites is reduced when imposing higher minimum coverage thresholds.

Supplementary Figure S5

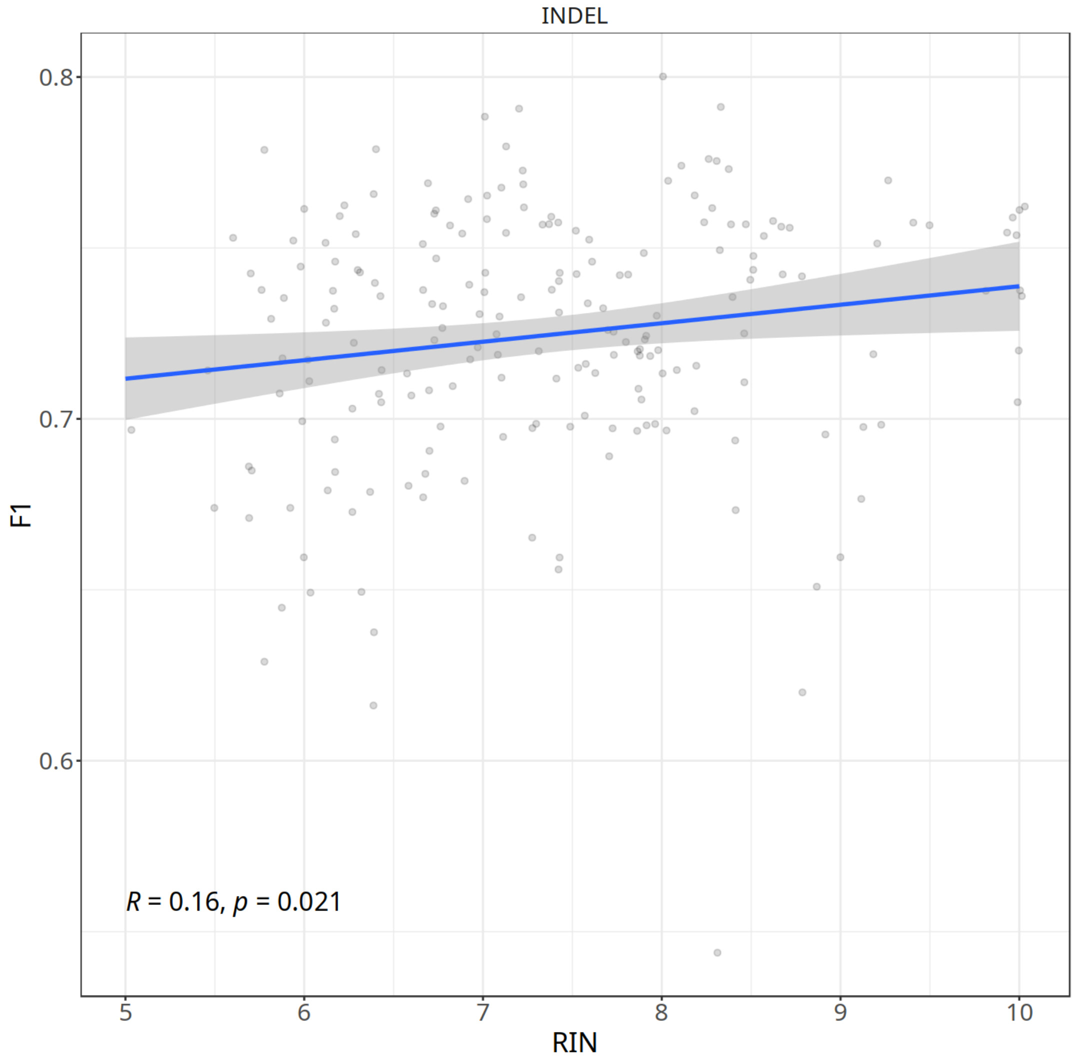

Supplementary Figure S5: Correlation between RIN and F1-score for INDELs in CDS regions. The correlation between RIN and the F1 score is shown for 200 RNA-seq samples for INDEL calls in CDS regions.

Supplementary Figure S6

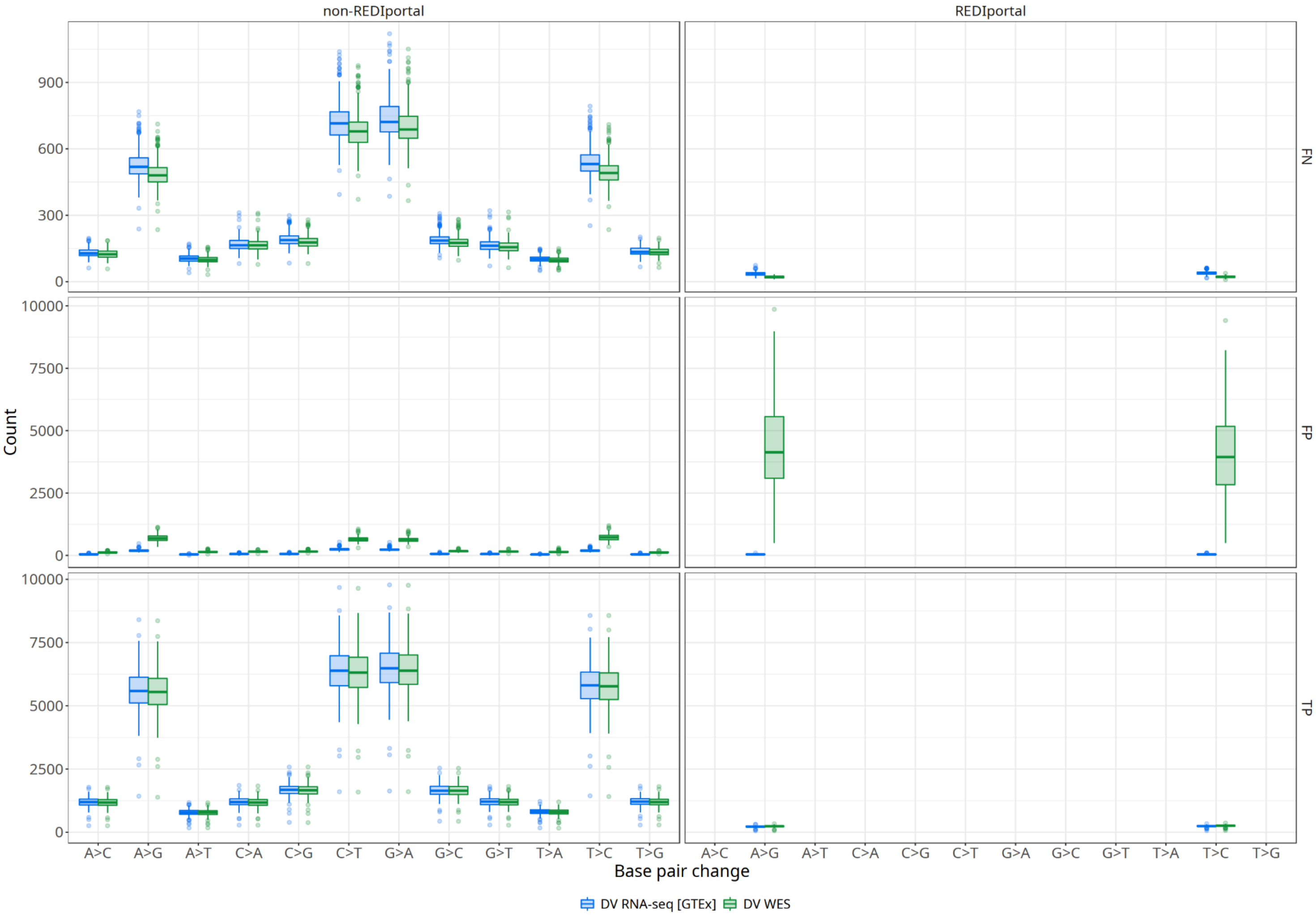

Supplementary Figure S6: Aggregate summary of RNA edited site classification. The distribution of false negative (FN), false positive (FP) and true positive (TP) events is shown by base change and whether or not a variant has previously been characterized as an RNA edit event in the REDIportal database. Results are shown for the DeepVariant RNA-seq and DeepVariant WES models.

Supplementary Figure S7

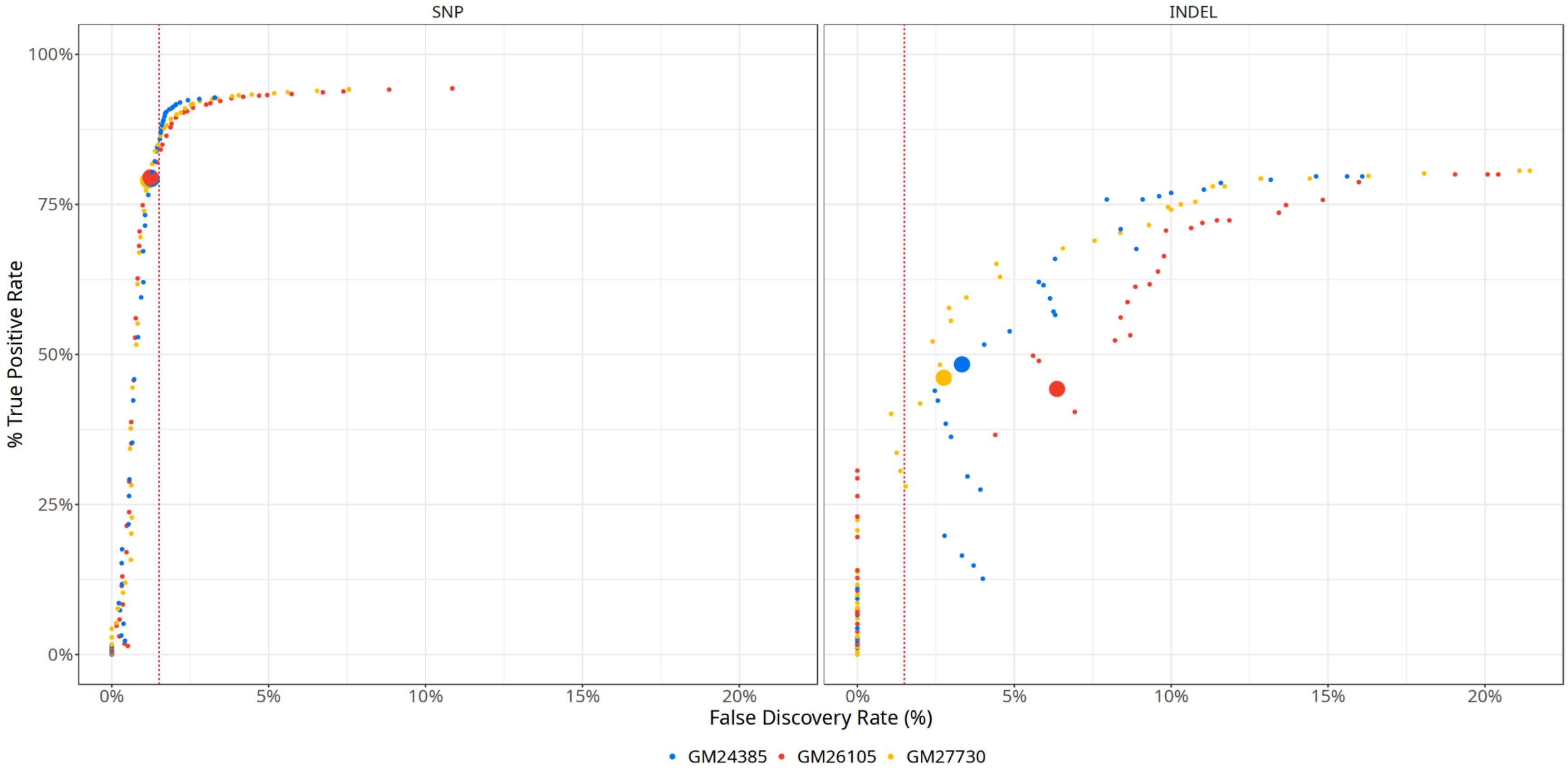

Supplementary Figure S7: Selection of a QUAL cut-off to maintain FDR of 1.5%. The FDR (x-axis) is plotted against the TPR (y-axis) for both SNP and INDELs across 3 HG002 cell lines. Each point corresponds with the FDR (x-axis) and TPR (y-axis) at a given QUAL cutoff. The red line indicates a FDR of 1.5%. Larger markers indicate a cutoff of QUAL>=25.

Supplementary Figure S8

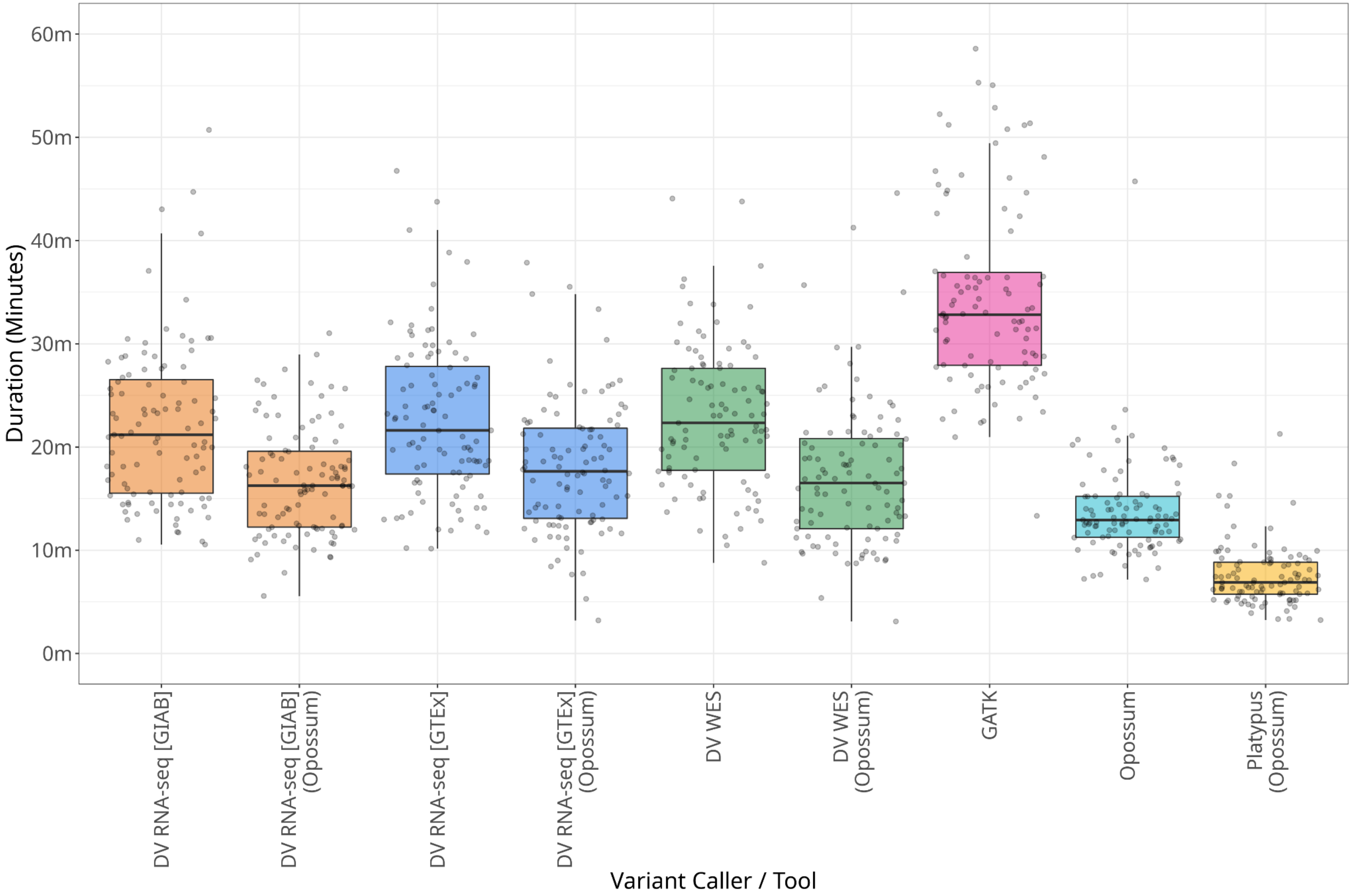

Supplementary Figure S8: Run time statistics. The runtime distribution of a random sample of 100 runs for each variant caller and Opossum are shown. Filtering was performed to remove excessive runtime outliers (>1 hr). These were only observed for GATK.
